# Resting-state functional network segregation of the default mode network predicts valence bias across the lifespan

**DOI:** 10.1162/imag_a_00403

**Published:** 2024-12-19

**Authors:** Jordan E. Pierce, Gagan S. Wig, Nicholas R. Harp, Maital Neta

**Affiliations:** Center for Brain, Biology, and Behavior, University of Nebraska-Lincoln, Lincoln, NE, United States; Department of Psychology, University of Nebraska-Lincoln, Lincoln, NE, United States; Center for Vital Longevity & Department of Psychology, University of Texas at Dallas, Dallas, TX, United States; Department of Psychiatry, University of Texas Southwestern Medical Center, Dallas, TX, United States; Department of Psychology, University of California, Berkeley, Berkeley, CA, United States

**Keywords:** valence bias, resting-state functional connectivity, lifespan, network segregation, default mode network

## Abstract

The brain is organized into intrinsically connected functional networks that can be reliably identified during resting-state functional magnetic resonance imaging (fMRI). Healthy aging is marked by decreased network segregation, which is linked to worse cognitive functioning, but aging-related changes in emotion are less well characterized. Valence bias, which represents the tendency to interpret emotionally ambiguous information as positive or negative, is more positive in older than younger adults and is associated with differences in task-based fMRI activation in the amygdala, prefrontal cortex, and a cingulo-opercular (CO) network. Here, we examined valence bias, age, and resting-state network segregation of 12 brain networks in a sample of 221 healthy individuals from 6 to 80 years old. Resting-state network segregation decreased linearly with increasing age, extending prior reports of de-differentiation across the lifespan. Critically, a more positive valence bias was related to lower segregation of the default mode network (DMN), due to stronger functional connectivity of the DMN with CO and, to a lesser extent, the ventral attention network (VAN) in all participants. In contrast to this overall segregation effect, in participants over 39 years old (who tend to show a positive valence bias), bias was also related to*weaker*connectivity between the DMN and Reward networks. The present findings indicate that specific interactions between the DMN, a task control network (CO), an emotion processing network (Reward), and, to a weaker extent, an attention network (VAN), support a more positive valence bias, perhaps through regulatory control of self-referential processing and reduced emotional reactivity in aging. The current work offers further insight into the functional brain network alterations that may contribute to affective well-being and dysfunction across the lifespan.

## 1 Introduction

The brain is organized into intrinsically connected functional networks that can be reliably identified during resting-state functional magnetic resonance imaging (fMRI;Fox et al., 2005;Snyder & Raichle, 2012). Despite being characterized at rest, these brain networks are also associated with task performance in relevant functional domains (Cohen & D’Esposito, 2016;Yeo et al., 2015). For example, the default mode network (DMN), which consists of regions in medial prefrontal, medial and lateral parietal and temporal cortex, is associated with functions including self-referential processing, autobiographical memory, and social cognition (Davey et al., 2016;Menon, 2023;Mevel et al., 2013;Spreng et al., 2009). Other commonly described networks include task control networks such as the cingulo-opercular (CO) and fronto-parietal networks (FPN), dorsal and ventral attention networks (DAN/VAN), and sensory networks such as the visual network (Damoiseaux et al., 2006;Dworetsky et al., 2021;Seitzman et al., 2020).

The interactions within and between these functional brain networks can be quantified using resting-state functional connectivity (RSFC; i.e., the correlation of the fMRI activation time series between brain regions). Within-network connectivity refers to the coactivation of nodes in a given network and is relatively strong, whereas between-network connectivity refers to the relationship between nodes of different networks and is generally weaker and more sparse (Sporns & Betzel, 2016;Wig, 2017). These measures can be further summarized as network (or system) segregation, which represents the relative balance of within- to between-network correlation strengths across the brain (Chan et al., 2014,^2018^; cf. modularity,Sporns & Betzel, 2016). Network segregation is an important property of brain organization that allows both specialization of function and cooperative activity; the brain needs to be moderately segregated in order to perform distinct functions efficiently, while remaining sufficiently integrated to communicate output across networks (Wig, 2017). The examination of network segregation provides insight into the brain’s higher order organization, complementing more focal analyses of specific network connectivity.

The organization of resting-state networks and their relationship with brain function change over the course of the human lifespan (Biswal et al., 2010). Organized RSFC emerges early in life and is refined during childhood and adolescence, though existing evidence of directional changes in segregation is mixed (Gao et al., 2015;Grayson & Fair, 2017;Satterthwaite et al., 2013). Specific networks may show distinct patterns of functional connectivity at different developmental stages that parallel sensorimotor or cognitive abilities (Grayson & Fair, 2017;Marek et al., 2016). On the other hand, during the course of healthy aging, lower network segregation is consistently observed in older adults, corresponding to a de-differentiation of the network, which is linked to worse cognitive functioning in multiple domains, including long-term memory and executive function (Chan et al., 2014;Chong et al., 2019;Geerligs et al., 2015;Koen et al., 2020). Despite evidence of cognitive decline in aging, fewer studies have explored connectivity as a function of age-related changes in emotion processing.

### 1.1 Valence bias, aging, and RSFC

In contrast to the effects observed for cognitive functions, emotional functions and network organization may be relatively spared in aging (Mather, 2016;Nashiro et al., 2017;Scheibe & Carstensen, 2010). Indeed, while cognitive networks consistently show lower segregation, emotion networks have shown maintained or even increased connectivity in later life (Cao et al., 2014;Malagurski et al., 2020;Nashiro et al., 2017). Behaviorally, as individuals age there is a shift toward positivity in emotional attention and memory, often paired with improvements in well-being (Mather, 2012;Mather & Carstensen, 2005). This positivity effect is also observed in one’s tendency to appraise ambiguously valenced stimuli (e.g., faces, scenes) as positive or negative (known as “valence bias,”Neta, 2024;Neta et al., 2009). Studies have shown that while valence bias is relatively negative in children and young adults (Neta & Whalen, 2010;Petro et al., 2018;Tottenham et al., 2013), it becomes less negative and more positive in older adults (Neta & Tong, 2016;Petro, Basyouni, et al., 2021;Shuster et al., 2017). The initial negativity hypothesis of valence bias proposes that ambiguous stimuli are automatically interpreted as negative in younger individuals, and that positive interpretations require an effortful, regulatory mechanism to overcome this initial appraisal (Harp, Gross, et al., 2024;Neta & Whalen, 2010;Neta et al., 2022;Pierce et al., 2023). In older adults, however, there appears to be a shift to default positivity, where positive interpretations can be generated more quickly with little regulatory control (Neta & Tong, 2016;Petro, Basyouni, et al., 2021). Task-based fMRI evidence has demonstrated that positivity in younger adults recruits regions of the prefrontal cortex (PFC) that are involved in cognitive reappraisal, in line with a regulatory mechanism (Petro et al., 2018;Pierce et al., 2023). Conversely, in older adults, positivity is associated with faster amygdala habituation in the absence of regulatory PFC activation, contributing evidence that a default positivity allows for resolution of emotional ambiguity with minimal top-down control (Petro, Basyouni, et al., 2021).

When considering ambiguity processing during early development, task-based connectivity between the amygdala and ventromedial PFC correlates with valence bias in children ages 6 to 13 years old—stronger regulation is associated with more positivity in children at a more advanced pubertal stage (Petro, Tottenham, et al., 2021). We also recently demonstrated that in children ages 6 to 17 years old, valence bias could be predicted from whole-brain RSFC using support vector regression, as well as from CO and amygdala networks together (Harp, Nielsen, et al., 2024). This study further showed that model prediction of valence bias was sensitive to the connectivity of the CO, FPN, Visual, and Subcortical networks. The CO network previously has been related to ambiguity processing (Neta, Nelson, et al., 2017), including in the valence bias task specifically (Pierce et al., 2023), providing performance reporting feedback (Gratton et al., 2017) that can shape subsequent emotional decision-making.

Collectively, these prior studies suggest that processing of emotional ambiguity in the valence bias task changes as individuals age, with differential contributions of emotion-sensitive regions such as the amygdala and regulatory regions within the PFC. These behavioral differences are also evident in patterns of functional activity. Stronger interactions between control and emotion processing regions might result in lower segregation if these networks are frequently coactivated during affective processing. While prior work has found that greater segregation was associated with better cognitive performance (Chan et al., 2014;Koen et al., 2020), in the emotion domain, relatively lower segregation (i.e., greater integration) might reflect more controlled processing that balances emotional reactivity. Either greater between-network connectivity (e.g., PFC regulation of amygdala) or weaker within-network connectivity (e.g., among emotion sensitive regions) could reduce the overall segregation of emotion networks, resulting in more integrated cognitive-emotion processing and, potentially, a more positive valence bias. To date, however, the relationship between network segregation/RSFC and behavioral patterns in the valence bias task across the lifespan has not been examined. Such findings could inform our understanding of lifespan differences in valence bias and emotion processing more broadly.

### 1.2 The current study

In the current work, we examined the relationship between RSFC, aging across the lifespan, and valence bias task performance. In a cross-sectional sample of healthy individuals aged 6 to 80 years old, we analyzed the effects of age on network segregation of 12 functional networks across the whole brain. We expected to replicate previous reports of lower segregation in older adults, and to extend this finding to a broader age range that includes children and adolescents. Most importantly, we investigated the relationship between segregation and valence bias to determine which networks may contribute to individual differences in emotion task performance across the lifespan. Based on previous resting-state and task-based fMRI studies using the valence bias task (Harp, Nielsen, et al., 2024;Petro et al., 2018;Pierce et al., 2023), we predicted that segregation and connectivity of the CO, FPN, Visual, and Reward networks may be critical to supporting task performance and/or sensitive to age-related changes in performance. Stronger connectivity of these networks with each other or additional networks may support positive interpretations of emotional ambiguity, such that lower segregation is associated with more integrated cognitive-emotion processing and a more positive bias. Given that valence bias has previously been related to mental health measures such as loneliness (Harp & Neta, 2023;Neta & Brock, 2021) and stress (Brown et al., 2017), the current findings can advance our understanding of the brain mechanisms supporting individual differences in emotional health in both development and adult aging.

## 2 Methods

### 2.1 Participants

Three hundred and forty-two participants were recruited from the Lincoln, Nebraska community and completed an initial pre-scanning session, including an assessment of valence bias. Participants had to be right-handed, have no history of neurological disorder, no current use of a psychotropic medication, and no MRI-incompatible metal implants. Of those who completed the first session, 12 participants were excluded for inaccurate responses in the valence bias task (M_age_= 17.08 years; see below) and were not invited back for the scanning session, 12 were excluded for failing to meet MRI compatibility criteria (M_age_= 37.33), and 20 opted out of the study (M_age_= 19.25). Of the 298 participants who returned for the scanning session, seven did not complete the resting-state scans (M_age_= 12.14), 24 were excluded due to a poor alignment between the imaging data and the atlas space (M_age_= 35.75), and 46 were excluded for having an inadequate amount of data retained after motion censoring (M_age_= 20.89; described below). The final sample included 221 participants (M(SD)_age_= 34.06 (21.06), range = 6–80; 139 female/82 male; 179 White, 19 more than one race, 11 Asian, 8 Black, 3 unknown race, 1 American Indian/Alaskan Native; and 194 not Hispanic/Latino, 22 Hispanic/Latino, 5 unknown ethnicity). All participants (and/or their legal guardian) confirmed understanding of the procedures, provided written informed consent, and received compensation for their participation. All procedures were approved by the local institutional review board.

### 2.2 Procedure

In a pre-scanning session, participants completed the valence bias task, followed by a series of questionnaires beyond the scope of the present report. As in previous work (Neta et al., 2009,^2013^), the valence bias task consisted of a two-alternative forced choice task in which images were categorized as either positive or negative and was administered in MouseTracker software (Freeman & Ambady, 2010). The task comprised four blocks (two faces, two scenes), each with 12 ambiguous and 12 clearly valenced trials (6 positive, 6 negative), for a total of 96 trials (48 ambiguous, 48 clearly valenced). The faces included 34 discrete identities taken from the NimStim (14 identities;Tottenham et al., 2009) and Karolinska Directed Emotional Faces (20 identities;Lundqvist et al., 1998) stimulus sets, displaying happy, angry, or surprised expressions. The scenes were selected from the International Affective Picture System (Bradley & Lang, 2007), and were previously validated as being emotionally ambiguous (Harp et al., 2021;Neta et al., 2013). The clearly positive and negative images were used as within-subjects controls to ensure task comprehension and compliance (as in prior work;Harp et al., 2021;Neta et al., 2019,^2022^), and participants with accuracy below 60% were not invited back for the scanning session. Valence bias was calculated as the percentage of positive categorizations for ambiguous faces and scenes.

Approximately 1 week after the initial session, eligible participants were invited to return for the scanning session. Functional scans included a passive face viewing task (Petro et al., 2018;Petro, Basyouni, et al., 2021), an emotion regulation task (Pierce, Blair, et al., 2022;Pierce, Haque, et al., 2022), and a resting-state scan, during which participants passively viewed a white crosshair on a black background. A subset of 63 children from the present study were also included in an alternate RSFC analysis using a different ROI set and modeling approach that focused on affect in development (Harp, Nielsen, et al., 2024).

### 2.3 Image acquisition

Data were collected on a Siemens 3T Skyra scanner housed within the Center for Brain, Biology, and Behavior at University of Nebraska-Lincoln. Structural images were collected using a T1-weighted MP-RAGE sequence (TR = 2.2 s, TE = 3.37 ms, slices = 192 interleaved, 1 mm isotropic voxel size, FOV = 256 mm, flip angle = 7^o^, total acquisition time = 5:07). Resting-state functional scans were collected using an EPI sequence (TR = 1.0 s, TE = 30 ms, slices = 51, voxel size = 2.5 mm isotropic, matrix = 84 x 84 mm, FOV = 210 mm, flip angle = 60^o^, multiband factor = 3). Resting-state scans were collected over one to three runs for a total of approximately 15 minutes.

### 2.4 Image preprocessing

Preprocessing steps included slice timing, correction for head movement within and across runs, and intensity normalization of the functional data via the T1-weighted scans. Each run was resampled in atlas space on an isotropic 3 mm grid combining movement correction and atlas transformation in a single interpolation (Harp, Nielsen, et al., 2024;Nielsen et al., 2019;Shulman et al., 2010). Structural and functional images were registered to a target atlas in Talairach space (Talairach & Tournoux, 1988) created from MP-RAGE scans of thirteen 7- to 9-year-old children and twelve 21- to 30-year-old adults scanned on a Siemens 3T MAGNETOM Trio scanner (TRIO_KY_NDC). After aligning the structural scans to the target atlas space, cortical reconstruction was completed in Freesurfer (Fischl, 2012).

### 2.5 Functional connectivity processing

Functional connectivity processing was conducted using in-house MATLAB scripts (Gratton et al., 2020;Nielsen et al., 2019;Power et al., 2012) and included demeaning and detrending of each functional run, regression of nuisance variables (i.e., global signal, cerebrospinal and white matter nuisance masks derived from Freesurfer, and six rigid-body motion parameters, motion derivatives, and Volterra expansion of motion estimate;Friston et al., 1996), frame censoring and interpolation of data within runs, a temporal band-pass filter (0.009 Hz <*f*< 0.08 Hz), and spatial smoothing (6 mm full width half maximum). Framewise displacement (FD) was calculated from preprocessing realignment estimates and then low-pass filtered to remove high-frequency noise (Gratton et al., 2020). Frames with greater than 0.2 mm FD were censored (removed) prior to analysis (Nielsen et al., 2019;Power et al., 2014). After framewise censoring, data segments with less than five contiguous frames were removed, as were any functional runs with fewer than 50 frames to ensure sufficient stability in the resting-state signal. Only participants with at least 800 remaining frames of resting data (13.3 minutes) were included in the analysis, and the first 800 frames (after motion exclusions) were selected from each participant to minimize the effects of data quantity on network measures (Han et al., 2024). In other words, the earliest volumes that did not contain excessive movement were included until the 800-frame limit was reached; later volumes were discarded. This cut-off was determined based on the distribution of retained frames in the current data (412–1,337 frames, median = 913) to balance retention of participants (82.8%) and retention of data within retained participants (84.4%).

### 2.6 Regions of interest

**Fig. 1.**
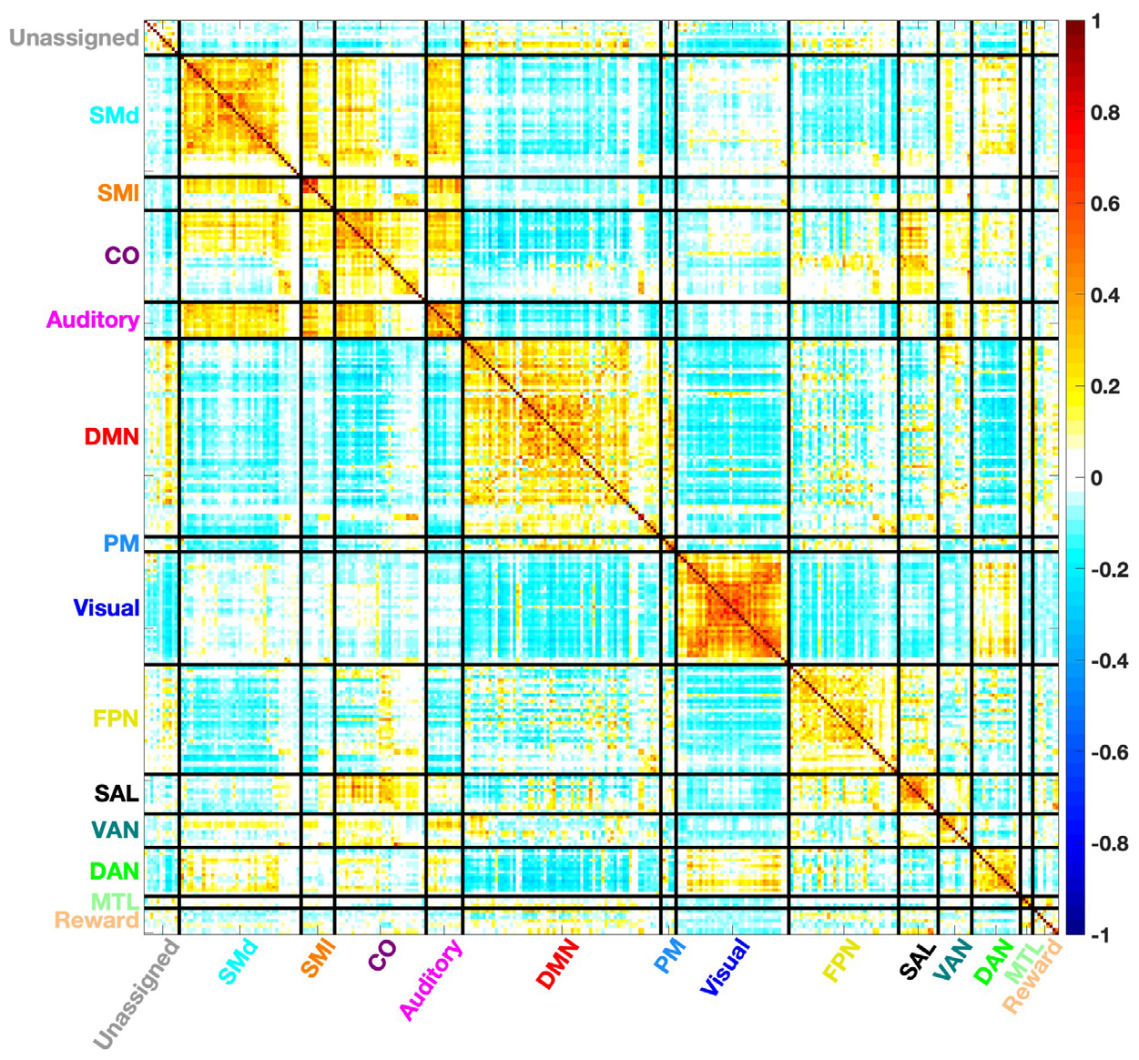
Average correlation matrix from all participants (n = 221) in 300 ROIs across 14 resting-state networks. On-diagonal blocks represent within-network connectivity, and off-diagonal blocks represent between-network connectivity.

RSFC time series were extracted from 300 whole-brain ROIs (5 mm radius;Seitzman et al., 2020). The time series from each ROI were correlated to produce a correlation matrix (Fig. 1), then normalized using a Fisher z-transform. This set of ROIs consists of 14 functional networks: Somatomotor-Dorsal (SMd), Somatomotor-Lateral (SMl), Cingulo-Opercular (CO), Auditory, Default Mode (DMN), Parietal Medial (PM), Visual, Fronto-Parietal (FPN), Salience (SAL), Ventral Attention (VAN), Dorsal Attention (DAN), Medial Temporal Lobe (MTL), Reward, and Unassigned. For network-level analyses, the Somatomotor-Dorsal and Somatomotor-Lateral networks were combined into a single network and the unassigned ROIs were excluded, leaving 12 networks.

### 2.7 Network (system) segregation

RSFC was analyzed according to the methods described byChan et al. (2014)for measuring within- and between-network functional connectivity (referred to here as just connectivity), which were combined into the single metric of network segregation. Briefly, within-network connectivity was defined as the mean correlation (z-value) of all ROIs within a given network to each other, and between-network connectivity was defined as the mean correlation of all ROIs in a given network to all other ROIs in the brain (or to all ROIs in each other network for network-level analyses). The segregation metric was defined as the difference in mean within- and mean between-network correlation as a proportion of mean within-network correlation (scripts available athttps://gitlab.com/wiglab/system-segregation-and-graph-tools) and represents the functional specialization of the brain network with respect to overall brain organization. As in prior work (Chan et al., 2014,^2018^;Zhang et al., 2023), all (unthresholded) positive correlations were included in the analysis, while all negative correlations were set to zero, given that global signal regression may introduce spurious negative correlations (Murphy et al., 2009). All networks (regardless of number of ROIs) were given equal weighting in calculating overall segregation; segregation was not correlated with the number of ROIs within each network (*r*= .20,*p*= .53). Each network, therefore, had three RSFC metrics entered into the analyses: 1) between-network connectivity, 2) within-network connectivity, and 3) network segregation.

### 2.8 Linear models

In the first analysis, we tested the relationship between age (standardized) and within-network connectivity, between-network connectivity, and segregation using linear models to identify linear or quadratic effects of age. The root mean square of FD per participant was regressed out of each RSFC measure to further control for any effects of motion (segregation and FD:*r*= -.45,*p*< .001; cf. FD as a covariate inChan et al., 2014,^2018^;Zhang et al., 2023). One subject was identified as an extreme outlier for segregation and removed from this analysis. Next, we explored the relationship between age and network measures for each individual network to determine if there were different patterns of maturation across the networks.*p*-Values were corrected using the false discovery rate (FDR) for the overall model fits to control for multiple comparisons across the 12 networks.

Secondly, linear models were fit predicting valence bias from segregation and, separately, from within-network and between-network connectivity (controlled for FD). Due to previous findings of broad changes in connectivity across the brain during aging (Chan et al., 2014;Geerligs et al., 2015;Nashiro et al., 2017), and an absence of evidence linking valence bias to patterns of large-scale connectivity in adults, we included all 12 functional networks in this analysis to evaluate segregation changes in relation to valence bias across multiple brain networks. Standardized age was included as a covariate, given the known changes in valence bias across the lifespan (Neta & Tong, 2016;Petro, Basyouni, et al., 2021;Shuster et al., 2017). Based on the results of this initial analysis (see Results), follow-up analyses were conducted on the between-network connectivity of the DMN to determine which other networks were contributing to the observed segregation effects (within-network connectivity was non-significant). Connectivity between the DMN and each of the 11 other networks was entered into a model predicting valence bias, along with age. Augmented backward elimination (R package: abe;Dunkler et al., 2014) was used for stepwise selection of variables based on the Akaike information criterion (AIC). Next, given the wide age range in our sample, we tested age as a moderator of the effects of DMN connectivity with the remaining selected networks; any age interaction that was not significant was not included in the final model. Network segregation and linear model analyses were conducted in R Statistical Software, version 4.3.1 (R Core Team, 2023).

## 3 Results

### 3.1 Segregation and age

**Fig. 2.**
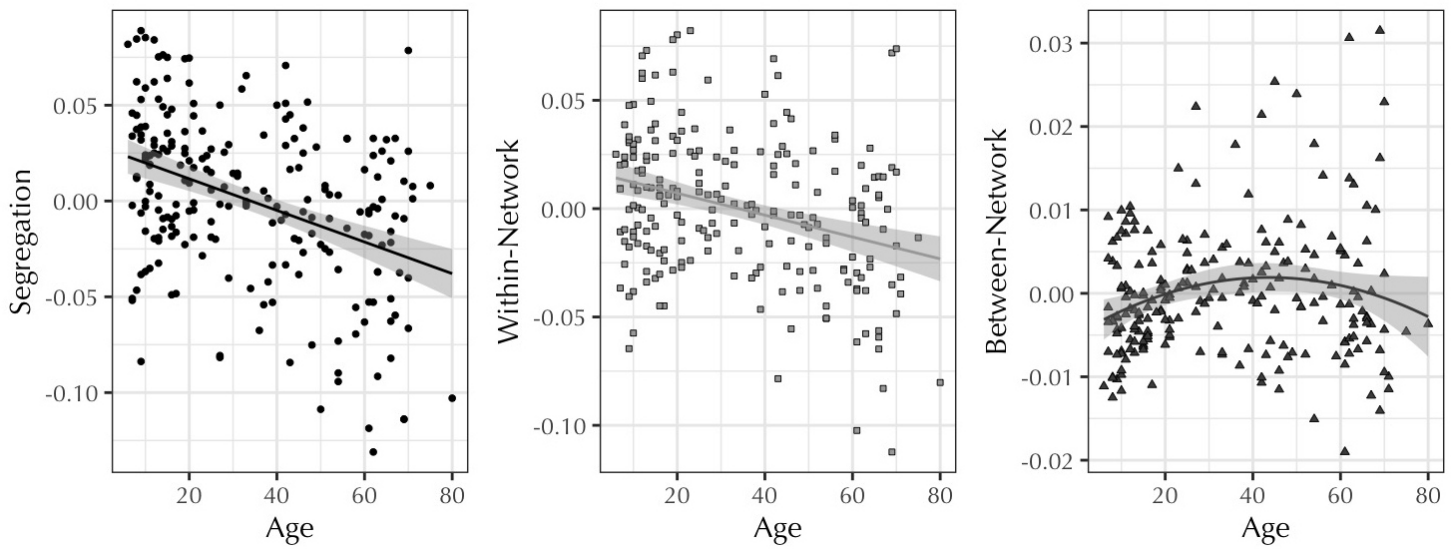
Relationships between segregation, within-network, and between-network connectivity and age. Values are residuals after controlling for FD. Segregation and within-network connectivity showed a negative linear effect with age, whereas between-network connectivity showed an inverted-U quadratic effect with age.

**Fig. 3.**
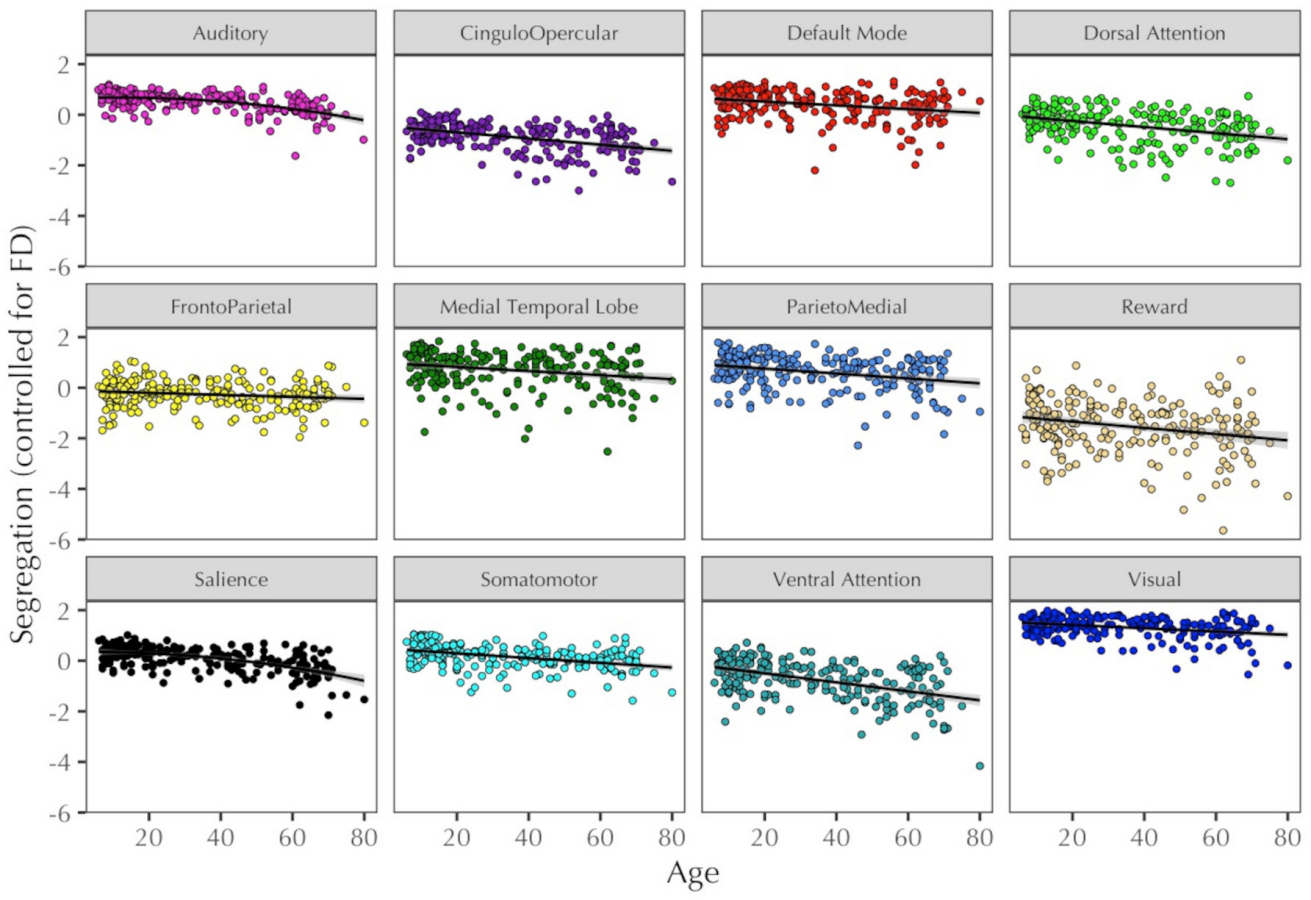
Relationship between segregation (controlled for FD) and age for each brain network. Trendlines show the significant quadratic effects for the Auditory and Salience networks and negative linear effects for all networks.

Linear models predicting segregation and within-network connectivity exhibited significant negative linear effects of age, such that segregation and within-network connectivity were lower in older participants. The linear model predicting between-network connectivity showed both a positive linear and negative quadratic effect of age, corresponding to an inverted U-shaped curve (Fig. 2;Table 1). Subsequently, the relationship between segregation and age was examined for each of the 12 resting-state networks separately. All networks exhibited significant negative linear effects of age on network segregation, and the Auditory and Salience networks also showed a significant quadratic effect of age (Fig. 3;Supplementary Table S1; for these effects separated by within- and between-network connectivity, seeSupplementary Tables S2 and S3, respectively).

**Table 1.** Models predicting network measures from age.

Model	*B*	SE	*t* -value	*p* -value
Segregation ( *F* (1, 218) = 41.11, *p* < .001, * R ^2^* = .159)				
Intercept	−0.0004	0.062	−0.007	.994
Age	−0.400	0.062	−6.412	<.001 **
Within-Network ( *F* (1, 218) = 23.54, *p* < .001, * R ^2^* = .098)				
Intercept	−0.0002	0.064	−0.003	.997
Age	−0.313	0.064	−4.852	<.001 **
Between-Network ( *F* (2, 217) = 4.03, *p* = .019, * R ^2^* = .036)				
Intercept	0.199	0.110	1.808	.072
Age	0.183	0.074	2.494	.013 *
Age (quadratic)	−0.200	0.088	−2.272	.024 *

**p< .01, *p< .05.

### 3.2 Valence bias and segregation

**Fig. 4.**
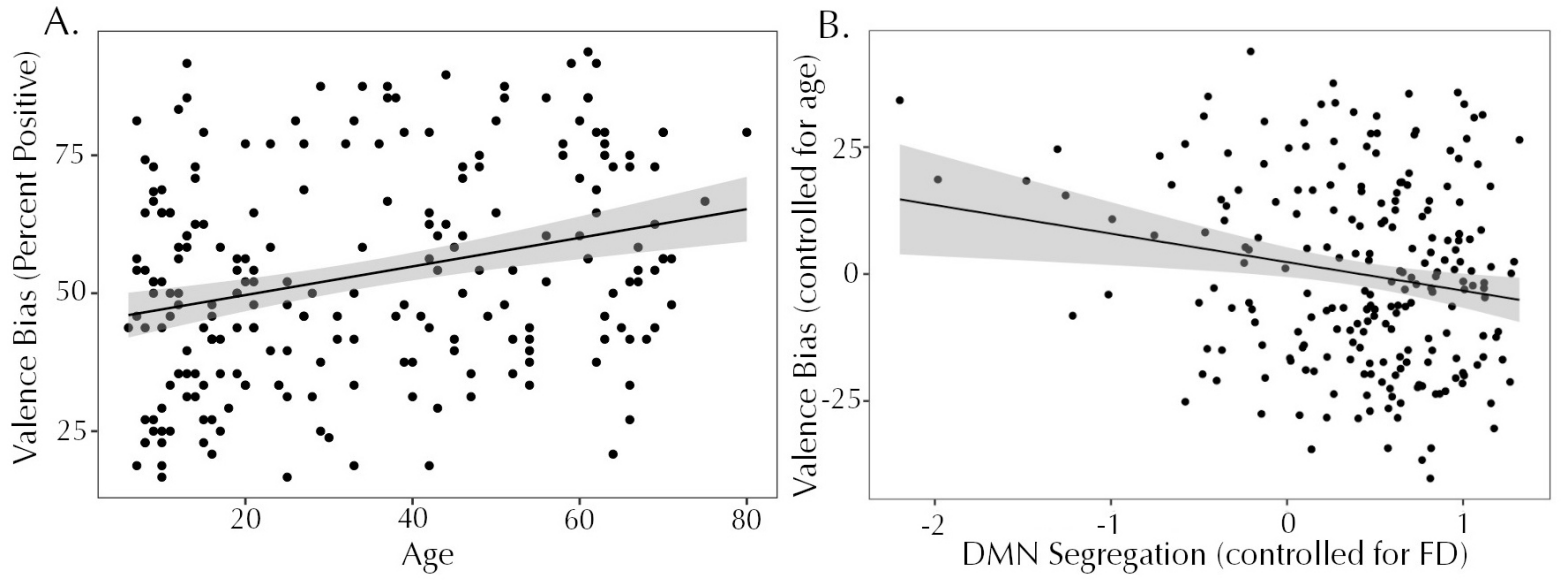
(A) Positive relationship between valence bias and age (*rho*= .256): older participants tended to have a more positive valence bias than younger participants. (B) Negative relationship between valence bias (controlled for age) and segregation (controlled for FD) of the DMN: lower network segregation was associated with a more positive valence bias.

Next, the relationship between segregation in each brain network and performance on the valence bias task was assessed. Valence bias was calculated as the percent of positive responses to ambiguous faces and scenes, and ranged from 16.67 to 93.75 with a median of 52.08 (SD = 19.06). Valence bias was positively correlated with age (*rho*= .256,*p*< .001, CI [.134, .379]), as expected, with older participants having a more positive valence bias (Fig. 4A). Given that age was associated with valence bias, it was included as a covariate in the linear models relating valence bias to segregation. Models for all networks were significant overall due to the age effects (Supplementary Table S4). Only in the DMN model (*F*(2, 218) = 14.04,*p*< .001,*R^2^*= .114), however, was the effect of segregation on valence bias significant, with lower segregation associated with a more positive bias (Fig. 4B). Models predicting valence bias from within- and between-network connectivity were also analyzed (Supplementary Table S5): only the DMN showed a significant effect of between-network connectivity (*F*(3, 217) = 8.76,*p*< .001,*R^2^*= .108), and no networks showed significant effects of within-network connectivity.

**Fig. 5.**
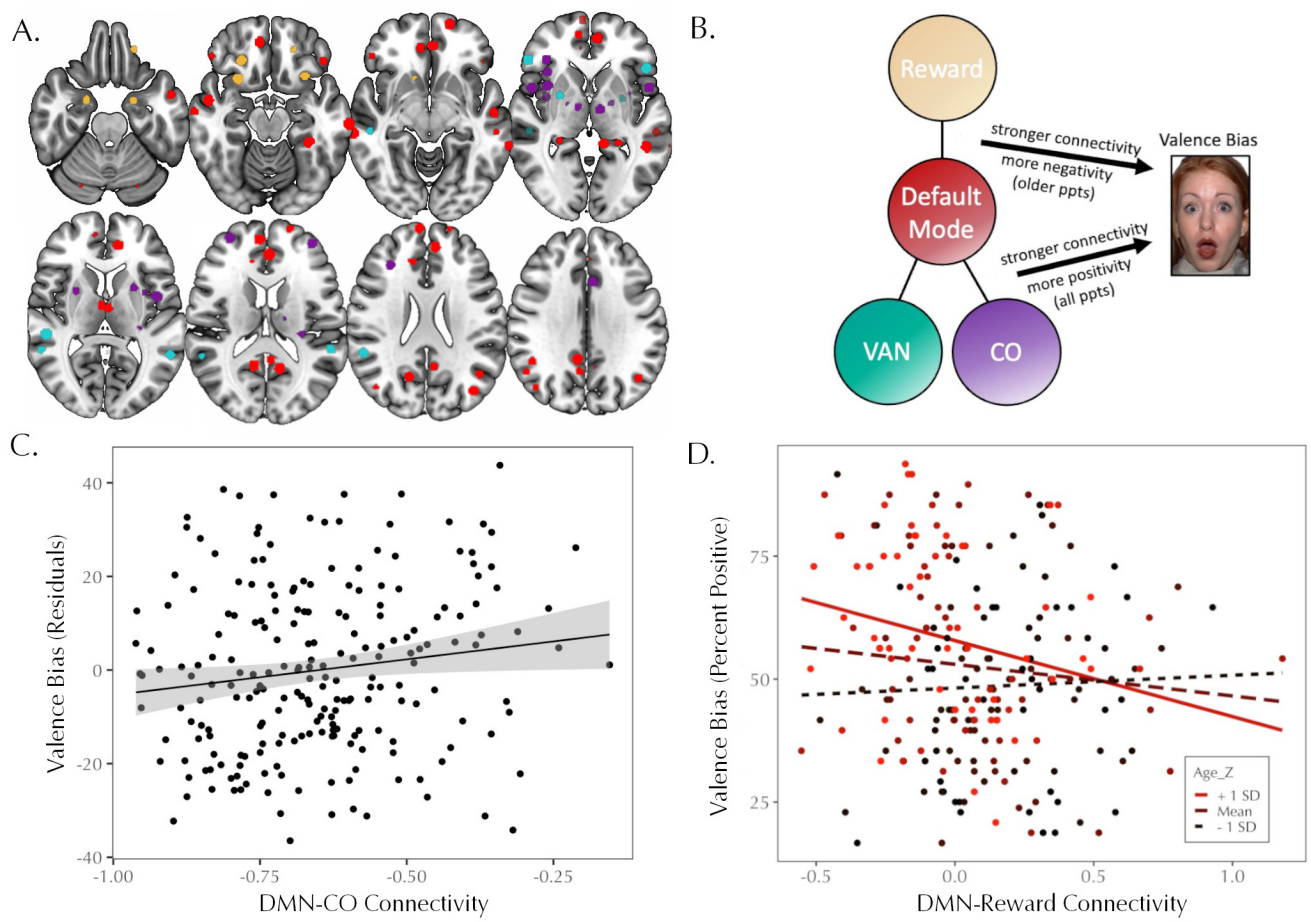
(A) Locations of the ROIs in the DMN (red), CO (purple), Reward (tan), and VAN (teal) networks. (B) Schematic summarizing the model predicting valence bias from DMN connectivity with the CO, Reward, and VAN networks. (C) Main effect of DMN-CO between-network connectivity on valence bias residuals (with two outliers removed). Stronger connectivity between the DMN and CO networks was associated with a more positive valence bias. (D) Interaction effect of age and DMN-Reward between-network connectivity. For older participants (>39.26 years; solid red line), weaker connectivity between the DMN and Reward networks was associated with a more positive valence bias.

Given the significant effect of between-network connectivity in the DMN model, we next explored which specific between-network connections were contributing to the relationship with valence bias. The initial model including all networks as predictors is shown inSupplementary Table S6; predictors were automatically removed in a stepwise manner to minimize the AIC of the model. The final model (*F*(5, 215) = 8.424,*p*< .001,*R*^2^= .164) included age, connectivity with the CO network, with the VAN network, and with the Reward network, and the interaction between age and Reward connectivity (Table 2). Connectivity between DMN and CO had a significant relationship with valence bias, such that stronger connectivity was associated with a more positive valence bias (*p*= .007). The effect of DMN and VAN connectivity was in a similar direction, but only reached trend-level significance (*p*= .053). The main effect of DMN and Reward connectivity was not significant, but its interaction with age was significant. For participants at one SD above the mean age (55.12 years), the simple slope of DMN-Reward connectivity with valence bias was significant (slope = -15.46,*t*= 2.615,*p*= .010); for participants at or younger than the mean age (34.06 years), this relationship was not significant (*p*> .05;Fig. 5). An analysis of the Johnson-Neyman interval indicated that for participants older than 39.26 years, there was a significant effect (*p*< .05), such that weaker connectivity between the DMN and Reward networks was associated with a more positive valence bias. Two potential outliers were evident in the DMN-CO connectivity values; the overall model (*F*(5, 213) = 7.536,*p*< .001,*R*^2^= .150) and effects described above all remained significant after excluding these two participants.

**Table 2.** Final model predicting valence bias from connectivity between DMN and individual networks.

Predictor	*B*	SE	*t* -value	*p* -value
Intercept	65.418	4.580	14.285	<.001 **
Age	4.819	1.285	3.749	<.001 **
DMN-CO	18.889	6.927	2.727	.007 **
DMN-VAN	7.609	3.904	1.949	.053 ^+^
DMN-Reward	−6.435	4.311	−1.493	.137
DMN-Reward * Age	−9.029	4.218	−2.141	.033 *

**p< .01, *p< .05,^+^*p*< .10.

## 4 Discussion

In this study, we examined RSFC within and between 12 functional networks in a sample of 221 healthy individuals from 6 to 80 years old. Across the lifespan, network segregation and within-network connectivity decreased, whereas between-network connectivity showed an inverted U-shape. Participants also completed a behavioral task assessing emotional judgments of ambiguous faces and scenes, yielding a measure of their tendency toward positive or negative appraisals, known as valence bias. As in previous work, valence bias was more negative in younger age and more positive in older age. Valence bias was negatively related to network segregation in the DMN, with contributions of the between-network connectivity with the CO, Reward, and VAN networks. Specifically, a more positive valence bias was associated with lower segregation of the DMN, stronger connectivity between the DMN and both the CO and VAN, and, in older participants, weaker connectivity between the DMN and the Reward network. Collectively, the present findings confirm prior reports of reduced segregation in aging and suggest that interactions, measured at rest, between the DMN, a control network (CO), an emotion processing network (Reward), and an attention network (VAN) support a more positive valence bias.

### 4.1 Network segregation decreases across the lifespan

The analysis of network segregation and age replicated previously reported effects in adulthood (e.g.,Chan et al., 2014;Geerligs et al., 2015), with lower overall segregation and within-network connectivity exhibiting a trend toward de-differentiation in older participants. We also showed that this pattern extended to children as young as 6 years old, with only the Auditory and Salience networks showing a quadratic effect where segregation was flat or increased slightly during childhood. On the other hand, between-network connectivity for all networks combined showed an inverted U-shape across age, with the strongest between-network connectivity in young adults. Some prior work has found increasing segregation in cognitive networks in childhood, interpreted as a refinement of associative functions over development (Satterthwaite et al., 2013;Wig, 2017), while other studies (e.g.,Marek et al., 2016) showed effects similar to the present findings with decreased segregation from childhood into young adulthood. Motion artifacts or insufficient/differing amounts of data may have contributed to some previous findings (Grayson & Fair, 2017;Han et al., 2024;Snyder, 2022), as children and older adults may have more difficulty lying still in the scanner for an extended time. Given the known impact of head motion on RSFC (e.g.,Parkes et al., 2018;Power et al., 2015), here we applied rigorous corrections for motion, censoring volumes for which the BOLD signal was likely contaminated by movement and matching the number of volumes that were analyzed for each participant. Even with these corrections, we still observed a correlation between movement and network segregation (greater FD was associated with lower segregation, even when accounting for age). FD, therefore, was also regressed out of network measures to account for any potential residual movement-related variance given the inclusion of children and older adults in our sample. As with other measures of RSFC, the sensitivity of segregation to head motion means age differences in movement may impact the results despite corrections during processing, and further research is necessary to corroborate the current pattern of network segregation in children.

### 4.2 Valence bias and the default mode network

The default mode was the only network to show a relationship between segregation and valence bias, with lower segregation associated with greater positivity in judgments of ambiguity. Relatively lower segregation, or greater integration, of the DMN means that in individuals with the tendency to see things in a more positive light, this network might be more connected with other networks or less cohesive within itself, as compared to those who view things more negatively. Breaking down the segregation effect into within- and between-network connectivity indicated that it was driven by connections between the DMN and other networks, rather than connections within the DMN. Such between-network connections support the integration of different brain functions and allow for flexible responding under varying task demands (Wig, 2017). DMN activity is associated with functions, including self-referential processing and autobiographical thoughts (Raichle, 2015), and disrupted RSFC of the DMN, particularly in medial PFC, is linked with emotion dysfunction (Greicius et al., 2007;Menon, 2011;Sheline et al., 2010). Lower segregation of this network in individuals with a more positive valence bias suggests a history of stronger interactions with other functional networks such that internally directed DMN functions may habitually be more integrated with regions supporting affective control. These interactions could reflect greater sensitivity to or influence on brain functions, including emotion processing and regulation. The analysis of the specific between-network correlations of the DMN offers further insight into these interactions.

Between-network connectivity in the DMN was further broken down to identify specific networks that were driving the effect on valence bias. Stronger connectivity between the DMN and CO networks was associated with a more positive valence bias. A prior study in young adults similarly found that more integration (lower segregation) of the DMN with control networks was associated with greater optimism (Moser et al., 2021, see alsoYankouskaya et al., 2022). This is also consistent with previous task-based work demonstrating a role for the CO network in response to ambiguity (Neta et al., 2014), and in particular, a regulatory role that supports decision-making during the valence bias task (Harp, Nielsen, et al, 2024,Neta et al., 2013;Pierce et al., 2023). Stronger connectivity between these networks could reflect a tendency to monitor and regulate ongoing self-referential thoughts, perhaps exploring alternate appraisals of daily experiences that are more goal-congruent (i.e., positively valenced).

Connectivity between the DMN and VAN also showed a marginal effect in the same direction, with stronger connectivity associated with more positivity. The VAN is typically associated with bottom-up reorienting of attention, including to emotional stimuli (Frank & Sabatinelli, 2012;Viviani, 2013;Vossel et al., 2014), and its involvement here may indicate that more positive individuals habitually experience differential allocation of attention (cf.Neta & Dodd, 2018). For example, positive interpretations of emotional stimuli may involve negative features capturing attention less strongly than positive features (Neta, Tong, et al., 2017;Singh et al., 2020;Todd et al., 2012), thus minimizing the intensity of one’s negative affective response or ruminative thoughts. Stronger connectivity with the DMN could indicate that the VAN is more sensitive to internal processing (Viviani, 2013;Wang et al., 2023;Whitfield-Gabrieli & Ford, 2012), perhaps including emotional reappraisals, than (negative) environmental cues (Harp, Gross, et al., 2024;Todd et al., 2012;Wadlinger & Isaacowitz, 2011), though this trend-level effect warrants further investigation.

Finally, we observed an effect on valence bias of connectivity between the DMN and Reward networks. The Reward network includes the amygdala, striatum, and orbitofrontal regions involved in emotion processing (Seitzman et al., 2020). Emotion-related structures and functions have been found to show less age-related decline than do cognitive functions, with a positivity effect in aging that is characterized by increased attention to and memory for positive emotions in older age (Mather, 2016;Scheibe & Carstensen, 2010;Teater & Chonody, 2020), and greater white matter integrity in frontal cortex (Viher et al., 2024). The interaction with age observed here indicated that the positivity effect in valence bias is associated with reduced connectivity between the DMN and emotion processing regions only for adults older than 39 years. This age-related effect is in the opposite direction of the overall segregation effect in the DMN and the DMN-CO connectivity described above, possibly reflecting differential contributions of regulatory versus emotion reactivity networks to DMN activity. This pattern of findings highlights the utility of probing the between- and within-network connectivity of systems rather than relying purely on the overall summary metric.

Older adults may be less reactive to (negative) emotional stimuli or less likely to dwell upon negative memories (Mather, 2012;Scheibe & Carstensen, 2010), allowing them to more easily adopt a positive appraisal of ambiguous stimuli. Moreover, there is evidence that older adults use distraction (switching attention) more frequently than cognitively demanding emotion regulation strategies, such as reappraisal (Martins et al., 2015;Mather, 2016;Scheibe et al., 2015). Speculatively, these different emotion processing tendencies could shape (or be shaped by) the connectivity between the DMN and Reward networks, further limiting the impact of negative stimuli on self-referential processing in more positive adults. Finally, it is worth noting that although research on aging often focuses on changes observed at more advanced ages (Mather, 2010), the present findings suggest that RSFC differences related to emotional positivity effects already emerge in middle-aged adults.

### 4.3 Limitations

The current findings should be considered with respect to several limitations. First, the network segregation metric only considered positive correlations, so negative correlations between nodes (which can be influenced by preprocessing choices such as global signal regression) did not contribute to the results. While this approach allows for better estimation of network coactivation, it means that inverse connectivity, such as that characterizing the downregulation of the amygdala by ventromedial PFC in emotion processing (Petro, Tottenham, et al., 2021;Sakaki et al., 2013), may be missed by the current approach. Differences in segregation should thus be interpreted as reflecting stronger versus weaker positive network connectivity, rather than negative correlations.

Secondly, the wide age range of participants in this sample means that anatomical changes across the lifespan may not be fully accounted for by the atlas registration (but seeHan et al., 2018). We used an atlas based on the brains of children and young adults, creating the possibility that the localization of ROIs in some older adults could be suboptimal. Thirdly, more children/younger participants were excluded based on their behavioral responses on the valence bias task (though this constituted only 3.5% of the initial sample). Although this task has been used in pediatric samples with success (Petro, Tottenham, et al., 2021;Tottenham et al., 2013), it does include stimuli that were normed using adult participants, so it is possible that some children interpreted the emotional content differently than the adults did or simply could not fully understand the task instructions. Finally, our sample consisted of participants who predominantly identified their race as White/European American, limiting the generalizability of these findings to individuals of other racial and ethnic backgrounds.

### 4.4 Future directions

The differences in network segregation observed in relation to valence bias have broader implications for how affective biases, such as those that may accompany mental health disorders, correspond to disruptions in the brain’s large-scale functional connectivity (Menon, 2011;Schwartz et al., 2019;Sheline et al., 2010;Spreng et al., 2020). A more negative valence bias has been related to increased loneliness (Harp & Neta, 2023), stress (Brown et al., 2017), anxiety (Park et al., 2016), and daily negative affect (Puccetti et al., 2023), while a more positive valence bias has been related to increased social connectedness (Neta & Brock, 2021). Prior work has also demonstrated that although valence bias is relatively stable within an individual over a period of 1–2 years (Harp et al., 2022), it is malleable: greater positivity was observed following an 8-week mindfulness intervention (Harp et al., 2022) and cueing of cognitive reappraisal within a single session (Neta et al., 2022). Considered with the current results, this raises the possibility that a shift in valence bias within an individual that impacts well-being could be accompanied by a reorganization of network RSFC. Future work could explore the relationship between valence bias, network segregation, and mental health following positivity-inducing interventions across the lifespan.

### 4.5 Conclusion

The present study demonstrated that age and individual differences in valence bias were associated with differences in segregation of resting-state brain networks. Notably, stronger functional connectivity between the DMN and both the CO and VAN networks was related to a more positive valence bias, suggesting a potential regulatory influence on self-referential processing that shapes emotional appraisals. Furthermore, age moderated an effect of DMN and Reward network connectivity on valence bias. In older participants (who tended to show greater positivity), weaker connectivity between these two networks was associated with a more positive valence bias, possibly indicating that a tendency for less emotional reactivity supports positivity. Collectively, these findings illustrate that emotional biases in the processing of ambiguously valenced stimuli are supported by differences in the organization of the brain’s intrinsic functional networks, offering further insight into the mechanisms that may contribute to affective well-being or dysfunction across the lifespan.

## Supplementary Material

Supplementary Material

## Data Availability

Raw data for participants up to the age of 35 years are available on the NIH Data Archive (https://nda.nih.gov/). Correlation matrices from all participants are publicly available on OSF (https://osf.io/hj5gv/). Network segregation scripts are available athttps://gitlab.com/wiglab/system-segregation-and-graph-tools.
