# Supplementary material for: Resting-state functional network segregation of the default mode network predicts valence bias across the lifespan
Source: Imaging Neurosci (Camb). 2024 Dec 19;2:imag-2-00403. doi: 10.1162/imag_a_00403 (PMC12315725; doi:10.1162/imag_a_00403)
Supplement: Supplementary Material [file imag_a_00403-supp.pdf]

Resting state functional network segregation of the default mode network predicts valence bias across the lifespan

Supplemental Material

Jordan E. Pierce, Gagan S. Wig, Nicholas R. Harp, Maital Neta

Table S1. Models predicting segregation from age by network.

Network	*B*	SE	*t*-value	*p*-value
**Auditory ($F(2, 218) = 47.11, p < .001, R^2 = .302$)**				
Intercept	0.586	0.037	15.789	< .001**
Age	-0.179	0.025	-7.234	< .001**
Age (quadratic)	-0.085	0.029	-2.902	.004**
**CO ($F(1, 219) = 54.06, p < .001, R^2 = .198$)**				
Intercept	-0.867	0.035	-24.935	< .001**
Age	-0.256	0.035	-7.353	< .001**
**DMN ($F(1, 219) = 16.37, p < .001, R^2 = .070$)**				
Intercept	0.415	0.039	10.733	< .001**
Age	-0.157	0.039	-4.046	< .001**
**DAN ($F(1, 219) = 36.55, p < .001, R^2 = .143$)**				
Intercept	-0.416	0.042	-9.978	< .001**
Age	-0.252	0.042	-6.046	< .001**
**FPN ($F(1, 219) = 5.05, p = .026, R^2 = .023$)**				
Intercept	-0.257	0.037	-6.882	< .001**
Age	-0.084	0.037	-2.247	.026*
**MTL ($F(1, 219) = 11.85, p < .001, R^2 = .051$)**				
Intercept	0.707	0.049	14.411	< .001**
Age	-0.169	0.049	-3.443	< .001**
**PM ($F(1, 219) = 20.83, p < .001, R^2 = .087$)**				
Intercept	0.620	0.045	13.850	< .001**
Age	-0.204	0.045	-4.564	< .001**
**Reward ($F(1, 219) = 13.44, p < .001, R^2 = .058$)**				
Intercept	-1.513	0.071	-21.394	< .001**
Age	-0.259	0.071	-3.667	< .001**
**SAL ($F(2, 218) = 53.43, p < .001, R^2 = .329$)**				
Intercept	0.165	0.046	3.612	< .001**
Age	-0.250	0.031	-8.167	< .001**
Age (quadratic)	-0.087	0.036	-2.392	.018*
**Somatomotor ($F(1, 219) = 43.63, p < .001, R^2 = .166$)**				
Intercept	0.162	0.030	5.431	< .001**
Age	-0.197	0.030	-6.605	< .001**
**VAN ($F(1, 219) = 64.64, p < .001, R^2 = .228$)**				
Intercept	-0.750	0.046	-16.20	< .001**
Age	-0.373	0.046	-8.04	< .001**
**Visual ($F(1, 219) = 25.01, p < .001, R^2 = .103$)**				
Intercept	1.322	0.027	48.394	< .001**
Age	-0.137	0.027	-5.001	< .001**

Bold font indicates a significant model fit (FDR-adjusted *p*-values).

$^{**}p < .01$, $^{*}p < .05$

Table S2. Models predicting *between-network* connectivity from age by network.

Network	B	SE	t-value	p-value
**Auditory (F(2, 218) = 6.61, p = .010, R^2 = .057)**				
Intercept	1.602	0.119	13.420	< .001**
Age	0.243	0.080	3.046	.003**
Age (quadratic)	-0.291	0.095	-3.067	.002**
CO (F(1, 219) = 0.89, p = .347, R^2 = .004)				
Intercept	0.519	0.040	12.959	< .001**
Age	-0.038	0.040	-0.942	.347
**DMN (F(2, 218) = 3.63, p = .048, R^2 = .032)**				
Intercept	-1.003	0.049	-20.423	< .001**
Age	0.057	0.033	1.740	.083⁺
Age (quadratic)	-0.101	0.039	-2.591	.010*
**DAN (F(1, 219) = 5.03, p = .048, R^2 = .022)**				
Intercept	0.240	0.042	5.760	< .001**
Age	0.093	0.042	2.243	.026*
FPN (F(2, 218) = 1.83, p = .217, R^2 = .017)				
Intercept	-0.312	0.050	-6.191	< .001**
Age	0.033	0.034	0.969	.334
Age (quadratic)	-0.076	0.040	-1.904	.058⁺
**MTL (F(2, 218) = 3.64, p = .048, R^2 = .032)**				
Intercept	-0.427	0.082	-5.193	< .001**
Age	0.135	0.055	2.461	.015*
Age (quadratic)	-0.132	0.065	-2.021	.045*
**PM (F(1, 219) = 5.121, p = .048, R^2 = .023)**				
Intercept	0.349	0.051	6.822	< .001**
Age	0.116	0.051	2.263	.025*
Reward (F(1, 219) = 3.23, p = .111, R^2 = .015)				
Intercept	-0.406	0.036	-11.151	< .001**
Age	-0.065	0.036	-1.797	.074⁺
SAL (F(1, 219) = 1.56, p = .232, R^2 = .007)				
Intercept	0.615	0.045	13.780	< .001**
Age	-0.056	0.045	-1.251	.212
**Somatomotor (F(2, 218) = 7.19, p = .010, R^2 = .062)**				
Intercept	0.374	0.079	4.730	< .001**
Age	0.180	0.053	3.419	< .001**
Age (quadratic)	-0.182	0.063	-2.905	.004**
VAN (F(2, 218) = 1.65, p = .232, R^2 = .015)				
Intercept	0.608	0.081	7.482	< .001**
Age	0.023	0.054	0.425	.671
Age (quadratic)	-0.115	0.065	-1.781	.076⁺
**Visual (F(1, 219) = 6.08, p = .048, R^2 = .027)**				
Intercept	-1.262	0.043	-29.595	< .001**
Age	0.105	0.043	2.467	.014*

Bold font indicates a significant model fit (FDR-adjusted p-values). ** $p < .01$, * $p < .05$, $^+p < .10$

Table S3. Models predicting *within-network* connectivity from age by network.

Network	*B*	SE	*t*-value	*p*-value
**Auditory ($F(2, 218) = 13.14, p < .001, R^2 = .108$)**				
Intercept	1.378	0.099	13.964	< .001**
Age	-0.137	0.066	-2.084	.038*
Age (quadratic)	-0.266	0.078	-3.403	< .001**
**CO ($F(1, 219) = 36.16, p < .001, R^2 = .142$)**				
Intercept	-0.606	0.022	-27.764	< .001**
Age	-0.131	0.022	-6.013	< .001**
**DMN ($F(1, 219) = 22.14, p < .001, R^2 = .092$)**				
Intercept	-0.455	0.024	-18.838	< .001**
Age	-0.114	0.024	-4.705	< .001**
**DAN ($F(1, 219) = 11.78, p < .001, R^2 = .051$)**				
Intercept	-0.417	0.032	-13.173	< .001**
Age	-0.109	0.032	-3.433	< .001**
**FPN ($F(1, 219) = 5.97, p = .017, R^2 = .027$)**				
Intercept	-0.618	0.022	-28.414	< .001**
Age	-0.053	0.022	-2.443	.015*
**MTL ($F(1, 219) = 5.32, p = .022, R^2 = .024$)**				
Intercept	0.525	0.081	6.521	< .001**
Age	-0.186	0.081	-2.307	.022*
**PM ($F(1, 219) = 12.63, p < .001, R^2 = .054$)**				
Intercept	0.935	0.077	12.209	< .001**
Age	-0.272	0.077	-3.554	< .001**
**Reward ($F(1, 219) = 13.53, p < .001, R^2 = .058$)**				
Intercept	-1.108	0.023	-47.815	< .001**
Age	-0.085	0.023	-3.679	< .001**
**SAL ($F(1, 219) = 62.92, p < .001, R^2 = .223$)**				
Intercept	0.150	0.035	4.272	< .001**
Age	-0.279	0.035	-7.932	< .001**
**Somatomotor ($F(1, 219) = 16.82, p < .001, R^2 = .071$)**				
Intercept	-0.0007	0.033	-0.021	.983
Age	-0.137	0.033	-4.101	< .001**
**VAN ($F(1, 219) = 34.97, p < .001, R^2 = .138$)**				
Intercept	-0.503	0.032	-15.844	< .001**
Age	-0.188	0.032	-5.914	< .001**
**Visual ($F(1, 219) = 17.90, p < .001, R^2 = .076$)**				
Intercept	0.987	0.055	17.844	< .001**
Age	-0.234	0.055	-4.231	< .001**

Bold font indicates a significant model fit (FDR-adjusted *p*-values).
**$p < .01$, *$p < .05$, $^+p < .10$

Table S4. Models predicting valence bias from segregation and age by network.

Network	*B*	SE	*t*-value	*p*-value
Auditory ($F(2, 218) = 9.97$, $p < .001$, $R^2 = .084$)				
Intercept	51.915	2.207	23.527	< .001**
Segregation	2.804	3.650	0.768	.443
Age	6.037	1.455	4.149	< .001**
CO ($F(2, 218) = 10.10$, $p < .001$, $R^2 = .085$				
Intercept	51.425	2.426	21.198	< .001**
Segregation	-2.182	2.405	-0.907	.365
Age	4.893	1.383	3.539	< .001**
**DMN ($F(2, 218) = 14.04$, $p < .001$, $R^2 = .114$)**				
**Intercept**	**55.826**	**1.505**	**37.100**	**< .001****
**Segregation**	**-6.041**	**2.127**	**-2.840**	**.005****
**Age**	**4.505**	**1.263**	**3.567**	**< .001****
DAN ($F(2, 218) = 9.68$, $p < .001$, $R^2 = .082$)				
Intercept	53.491	1.496	35.758	< .001**
Segregation	0.419	2.010	0.208	.835
Age	5.557	1.340	4.147	< .001**
FPN ($F(2, 218) = 9.82$, $p < .001$, $R^2 = .083$)				
Intercept	52.998	1.367	38.768	< .001**
Segregation	-1.243	2.243	-0.554	.580
Age	5.347	1.254	4.264	< .001**
MTL ($F(2, 218) = 10.48$, $p < .001$, $R^2 = .088$)				
Intercept	54.806	1.725	31.764	< .001**
Segregation	-2.107	1.704	-1.237	.217
Age	5.096	1.269	4.014	< .001**
PM ($F(2, 218) = 9.77$, $p < .001$, $R^2 = .082$)				
Intercept	53.860	1.698	31.718	< .001**
Segregation	-0.875	1.873	-0.467	.641
Age	5.273	1.298	4.063	< .001**
Reward ($F(2, 218) = 9.86$, $p < .001$, $R^2 = .083$)				
Intercept	52.208	2.179	23.964	< .001**
Segregation	-0.733	1.184	-0.619	.537
Age	5.261	1.277	4.120	< .001**
SAL ($F(2, 218) = 9.75$, $p < .001$, $R^2 = .082$)				
Intercept	53.414	1.262	42.333	< .001**
Segregation	-1.239	2.979	-0.416	.678
Age	5.104	1.494	3.416	< .001**
Somatomotor ($F(2, 218) = 10.14$, $p < .001$, $R^2 = .085$)				
Intercept	53.745	1.319	40.757	< .001**
Segregation	-2.643	2.807	-0.942	.347
Age	4.931	1.356	3.637	< .001**
VAN ($F(2, 218) = 9.76$, $p < .001$, $R^2 = .082$)				
Intercept	52.717	1.838	28.678	< .001**
Segregation	-0.799	1.808	-0.442	.659

Age	5.153	1.411	3.651	< .001**
Visual ($F(2, 218) = 10.38$, $p < .001$, $R^2 = .087$)				
Intercept	48.655	4.229	11.505	< .001**
Segregation	3.528	3.060	1.153	.250
Age	5.933	1.306	4.544	< .001**

Bold font indicates the model has a significant effect of segregation.

**$p < .01$, *$p < .05$

Table S5. Models predicting valence bias from between- and within-network connectivity by network.

**Network**	***B***	**SE**	***t*-value**	***p*-value**
Auditory ($F(3, 217) = 6.58, p < .001, R^2 = .083$)				
Intercept	52.233	2.008	26.016	< .001**
Between-network	0.103	1.970	0.052	.959
Within-network	0.856	2.376	0.360	.719
Age	5.633	1.472	3.826	< .001**
CO ($F(3, 217) = 6.81, p < .001, R^2 = .086$)				
Intercept	49.080	4.359	11.261	< .001**
Between-network	2.798	2.777	1.007	.315
Within-network	-4.593	5.091	-0.902	.368
Age	4.953	1.380	3.589	< .001**
**DMN ($F(3, 217) = 8.76, p < .001, R^2 = .108$)**				
**Intercept**	**58.595**	**3.835**	**15.278**	**< .001****
**Between-network**	**6.115**	**2.768**	**2.209**	**.028***
**Within-network**	**-3.234**	**3.466**	**-0.933**	**.352**
**Age**	**4.950**	**1.286**	**3.850**	**< .001****
DAN ($F(3, 217) = 6.72, p < .001, R^2 = .085$)				
Intercept	53.688	2.170	24.746	< .001**
Between-network	-1.799	2.484	-0.724	.470
Within-network	-0.146	3.267	-0.045	.964
Age	5.604	1.348	4.157	< .001**
FPN ($F(3, 217) = 7.30, p < .001, R^2 = .092$)				
Intercept	55.585	2.763	20.116	< .001**
Between-network	4.050	2.748	1.474	.142
Within-network	1.129	3.892	0.290	.772
Age	5.486	1.254	4.374	< .001**
MTL ($F(3, 217) = 7.43, p < .001, R^2 = .093$)				
Intercept	54.182	1.865	29.050	< .001**
Between-network	-0.073	1.838	-0.039	.969
Within-network	-1.723	1.148	-1.500	.135
Age	5.138	1.276	4.025	< .001**
PM ($F(3, 217) = 6.57, p < .001, R^2 = .083$)				
Intercept	53.306	1.645	32.411	< .001**
Between-network	1.070	1.692	0.632	.528
Within-network	-0.387	1.129	-0.343	.732
Age	5.222	1.306	3.999	< .001**
Reward ($F(3, 217) = 6.49, p < .001, R^2 = .082$)				
Intercept	52.743	4.231	12.466	< .001**
Between-network	1.133	2.457	0.461	.645
Within-network	-0.934	3.864	-0.242	.809
Age	5.446	1.282	4.249	< .001**
SAL ($F(3, 217) = 7.59, p < .001, R^2 = .095$)				
Intercept	51.089	1.743	29.309	< .001**

Between-network	4.100	2.366	1.733	.085$^+$
Within-network	-1.951	3.000	-0.650	.516
Age	5.135	1.452	3.538	< .001**
Somatomotor (F(3, 217) = 7.22, p < .001, R^2 = .091)				
Intercept	52.768	1.296	40.712	< .001**
Between-network	2.856	2.018	1.415	.158
Within-network	-3.484	2.960	-1.177	.241
Age	4.642	1.360	3.412	< .001**
VAN (F(3, 217) = 7.31, p < .001, R^2 = .092)				
Intercept	50.176	2.826	17.754	< .001**
Between-network	3.418	2.181	1.567	.119
Within-network	-2.889	3.410	-0.847	.398
Age	4.966	1.382	3.592	< .001**
Visual (F(3, 217) = 7.55, p < .001, R^2 = .095)				
Intercept	48.643	3.036	16.022	< .001**
Between-network	-3.381	1.964	-1.721	.089$^+$
Within-network	0.413	1.514	0.272	.786
Age	5.904	1.296	4.556	< .001**

Bold font indicates the model has a significant effect of connectivity.
**p < .01, *p < .05, ^+p < .10

Table S6. Initial model predicting valence bias from connectivity between the DMN and individual networks.

**Predictor**	***B***	**SE**	***t*-value**	***p*-value**
Intercept	62.419	8.477	7.363	< .001**
Age	4.139	1.358	3.048	.003**
DMN-Somatomotor	4.530	11.129	0.407	.684
DMN-CO	16.402	11.350	1.445	.150
DMN-Auditory	0.511	8.375	0.061	.952
DMN-PM	-1.900	2.475	-0.768	.444
DMN-Visual	-2.373	8.614	-0.275	.783
DMN-FPN	-1.605	5.385	-0.298	.766
DMN-SAL	2.152	6.412	0.336	.738
DMN-VAN	7.714	4.243	1.818	.071^{+}
DMN-DAN	-7.551	10.070	-0.750	.454
DMN-MTL	-1.205	2.966	-0.406	.685
DMN-Reward	-6.113	5.423	-1.127	.261

Full model: ($F(12, 208) = 3.15$, $p < .001$, $R^2 = .154$).
**$p < .01$, *$p < .05$, $^{+}p < .10$
